# Impact of COVID-19 pandemic on older cancer patients: Proposed solution by the International Geriatric Radiotherapy Group

**DOI:** 10.3389/fonc.2023.1091329

**Published:** 2023-03-07

**Authors:** Nam Phong Nguyen, Ulf Lennart Karlsson, David Lehrman, Thandeka Mazibuko, Tatul Saghatelyan, Juliette Thariat, Brigitta G. Baumert, Vincent Vinh-Hung, Olena Gorobets, Huan Giap, Sankalp Singh, Alexander Chi, Graciana Alessandrini, Abhinav Ahluwalia, Francis Durosinmi-Etti, Jorge Zegarra Cárdenas, Koniba Diabate, Joan Oboite, Eromosele Oboite, Tahir Mehmood, Te Vuong, Lyndon Kim, Brandi R. Page

**Affiliations:** ^1^ Department of Radiation Oncology, Howard University, Washington, DC, United States; ^2^ Department of Radiation Oncology, International Geriatric Radiotherapy Group, Washington, DC, United States; ^3^ Department of Radiation Oncology, National Center of Oncology, Yerevan, Armenia; ^4^ Department of Radiation Oncology, Francois Baclesse Cancer Center, Cain, France; ^5^ Institute of Radiation Oncology, Cantonal Hospital Graubuenden, Chur, Switzerland; ^6^ Department of Radiation Oncology, Centre Hospitalier de La Polynesie Francaise, Tahiti, French Polynesia; ^7^ Department of Oral Surgery, Centre Hospitalier Universitaire de Martinique, Martinique, France; ^8^ Department of Radiation Oncology, Medical University of South Carolina, Charleston, SC, United States; ^9^ Department of Radiation Oncology, Army Hospital (Research & Referral), New Delhi, India; ^10^ Department of Radiation Oncology, Beijing Chest Hospital, Beijing, China; ^11^ Department of Geriatry, Hospital Aleman, Buenos Aires, Argentina; ^12^ Department of Radiation Oncology, Advanced Care Oncology Center, Dubai, United Arab Emirates; ^13^ Department of Radiation Oncology, University of Lagos, Lagos, Nigeria; ^14^ Division of Medical Oncology, Regional Institute of Neoplastic Disease, Concepcion, Peru; ^15^ Radiotherapy Service at Bamako Hospital, Bamako, Mali; ^16^ Department of Radiation Oncology, Northampton General Hospital, Northampton, United Kingdom; ^17^ Department of Radiation Oncology, McGill University, Montreal, QC, Canada; ^18^ Division of Neuro-Oncology, Mount Sinai Hospital, New York, NY, United States; ^19^ Department of Radiation Oncology, Johns Hopkins University, Baltimore, MD, United States

**Keywords:** older, cancer patients, pandemic, fear, telemedicine, spiritual treatment

## Abstract

Older cancer patients are disproportionally affected by the Coronavirus 19 (COVID-19) pandemic. A higher rate of death among the elderly and the potential for long-term disability have led to fear of contracting the virus in these patients. This fear can, paradoxically, cause delay in diagnosis and treatment that may lead to a poor outcome that could have been prevented. Thus, physicians should devise a policy that both supports the needs of older patients during cancer treatment, and serves to help them overcome their fear so they seek out to cancer diagnosis and treatment early. A combination of telemedicine and a holistic approach, involving prayers for older cancer patients with a high level of spirituality, may improve vaccination rates as well as quality of life during treatment. Collaboration between health care workers, social workers, faith-based leaders, and cancer survivors may be crucial to achieve this goal. Social media may be an important component, providing a means of sending the positive message to older cancer patients that chronological age is not an impediment to treatment.

## Introduction

Coronavirus Disease 19 (COVID 19) is a pandemic of unprecedented and epic proportion which affects older patients disproportionally. A high rate of hospitalization and death has been reported among adults over 65 years of age (1). Among older patients who survived the infection, long-term complications such as dementia has been reported (2). Delirium during the viral infection may lead to later brain damage in this vulnerable population (3). Other studies also corroborated a higher rate of respiratory failure, fatigue, and dementia in older patients due likely to pre-existing comorbidities (4). Older cancer patients in particular, face compounded risk. They are likely to suffer a higher risk of severe illness, intensive care unit admission, mechanical ventilation, and death compared to non-cancer patients (5). Up to 28% of cancer patients admitted for COVID-19 infection died during hospital stay due to advanced age and multiple comorbidities such as high blood pressure and cardiovascular disease (6). Patients with hematologic malignancies are at a higher risk of death compared with patients with other solid tumors (7, 8). Other studies have corroborated the high mortality rate associated with old age, cancer, and COVID-19 infection (9). Thus, it is not surprising that patient fear of exposure to the virus has led to delayed diagnosis of cancer due to cancellation of diagnostic imaging procedures such as mammography or colonoscopy (10). Even though fear of virus infection was felt across the general population during the pandemic, its impact was greatest among older patients leading to anxiety and depression (11–13). Cancer patients undergoing active treatment also experienced a high level of emotional distress about the risk of infection (14). As a result, delay or avoidance of medical care increases significantly during the pandemic in the US and across the world (15). This behavior may have led to increased mortality among older cancer patients and minorities who already experience significant challenges in obtaining quality care in a reasonable time frame (16). As an international organization devoted to the care of older cancer patients, women, and minorities, the International Geriatric Radiotherapy Group (http://www.igrg.org) would like to propose a practical health care policy to support these vulnerable patients during the pandemic.

## Pandemic fear of infection among older cancer patients

Older patients, who at higher risk for death if infected with SARS-CoV-2, expressed fear during the COVID-19 pandemic across the globe. Using different testing scales such as the five-point fear of COVID-19 scale (FCV-19S), 44% of adults age 65 or older (n=500) admitted to fear of being exposed to the virus (12). This group of patients had preexisting comorbidities and were taking one to three medications at the survey time. Even though 98% of participants were fit, 35% suffered from loneliness, and depression. Those with a history of coronary artery disease, congestive heart failure, and chronic obstructive pulmonary disease expressed the worst fear of the virus which was compounded by social media news. In another study of 843 adults of at least 60 years of age, 28% expressed the fear of losing their lives to the virus. Their anxiety level was proportional to their age. Those who were 80 years-old or older expressed the worst fear (13). Fear of the virus is universal among older adults across the world, affecting all ethnicities (12, 17–21). The media plays a significant role in exacerbating older adults’ fear (20). There was a significant correlation between fear, insomnia, and depression likely due to catastrophizing, a negative thinking style characterized by the expectation of the worst possible outcome within a situation (20–22). As a result, in one study, 81% of the participants expressed moderate to severe depression leading to a high rate of insomnia (19). Among different ethnic groups in the United States, older African Americans developed the highest fear level likely related to the odds of death if infected (23–25). Concerns about contracting the disease and fear of racial bias in testing and treatment likely compound the anxiety (26). Thus, any public policy should take into consideration the ethnic diversity and the role of socio-economic inequalities in coping with the pandemic, and provide measures that address their concerns.

Older cancer patients in particular, face dual risk. Decreased immunity, advanced age, and comorbidities significantly increase the risks of death after contracting the virus (7, 27–29). Patients with hematologic malignancies are at higher risk of death compared with those with other types of cancer (7, 29). Thus, specific recommendations are needed for this type of malignancy. Regardless of the type of cancer, patients are concerned that coronavirus infection is a more life-threatening condition than cancer. As a result, cancer patients express fear and anxiety about the risk of virus exposure and the consequences of systemic treatment on their already altered immune system (30–33). In a survey, 66% of cancer patients were alarmed by the wide community spread of COVID-19, and this was the top cause of their anxiety (30). The fear of death is compounded by the anxiety of dying alone if infected for both patients and their loved ones (31–33). In light of these fears, it is understandable that older cancer patients expressed the highest level of anxiety and depression among those surveyed (34).

## Impact of COVID-19 on early cancer diagnosis

The fear of coronavirus infection has led to a significant decrease in cancer diagnosis. In a study of 82 patients with suspected breast cancer diagnosed on mammography, the rates of patients declining biopsy was 9.3% and 35.9% before and after the pandemic, respectively (35). The reason offered for refusal of the procedure was the fear of getting infected during the pandemic. In another study, the number of radiographic exams was significantly reduced during the pandemic compared with previous years, in an area affected by the pandemic. The number of radiographic exams performed was 211 in 2020 and ranged from 360 to 390 in the previous years (36). The combination of delayed biopsy procedures and radiographic exams has led to a precipitous decline in cancer diagnosis. For example, pathological diagnosis during the first six months of 2020 was reduced by 22.7% compared with the same time frame during the previous three years (37). Decreases were seen in the absolute number of pathologic specimen from all cancer sites but the lung, prostate, and gynecologic malignancies showed the steepest decline. Those three types of cancer are diagnosed with clinical exams, blood tests like PSA level, PAP smears, and radiographic exams which suggests a decline in outpatient clinic visits and/or hospital visits. Indeed, there was a significant reduction in the number of hospital visits and diagnostic procedures performed, including biopsy, colonoscopy, and mammography (38). The cancer-related hospitalization rate was reduced by 21%, and admissions were significantly reduced for all age groups. The reduction of procedures performed was 29%, 57%, and 55% for biopsies, colonoscopies, and mammograms, respectively. Other studies also corroborated a significant delay or decline in cancer diagnosis during the pandemic that was linked to a reduction in outpatient dental visits for oral cancer, and outpatient medical visits, for breast, prostate, and non-metastatic cancers, as well as various other types of cancer (39–45). Even though the decrease in cancer diagnosis was observed across all ages, individuals aged 64 or above were the most affected (42). Comparing the age groups 64 and older and younger than 64, reduction in cancer diagnosis was 35.2% and 25.2%, respectively. Another study also corroborated the significant decline in cancer diagnosis in older persons. In a multi-center cohort of 30 hospitals with specialization in the treatment of gastrointestinal cancers, the rate of cancer diagnosis in patients aged 65 or older was reduced to 42.4% and 17% during the lockdown and post lockdown period, respectively (43). This reduction in diagnosis of non-metastatic cancer in older patients is particularly worrisome because the prevalence of cancer increases with age. Early cancer diagnosis is critical to improving the survival of those vulnerable individuals with multiple comorbidities.

Zooming out to a global perspective, in Asia, delayed cancer diagnosis is often compounded by health inequalities between different socio-economic strata. In lower socio-economic strata, families are often overcrowded, with many generations living together. Older patients are at high risk of viral infection as younger family members are exposed to the virus while working full time during the pandemic (a necessity when under economic pressure). In addition, reallocation of health care workers and resources toward COVID-19 duties has further overwhelmed the already fragile health system (46). Thus, with the onset of the pandemic, cancer screening procedures were either completely stopped or reduced to less than 25%. The number of cancer diagnosis was reduced to 54% (47). **Table 1** summarizes cancer diagnosis delay during the pandemic.

**Table 1:** Decrease in cancer diagnosis during COVID-19 pandemic.

Study	Patient No.	Anatomic Site	Pre-COVID	Post-COVID	Reason	Country
Vanni et al. (35)	82	breast	90.7%	64.1%	Biopsy refusal	Italy
Hamilton et al. (37)	8168	all sites	4607	3561	unclear	Multiple
Fonseca et al. (38)	3, 261, 659	all sites	2, 471, 199	790, 460	Complex but virus Fear is a component	Brazil
Arduino et al. (39)	48	Oral cancer	40	8	travel restriction possible fear	Italy
Blay et al. (40)	91, 106	all sites	46, 802	44, 938	possible fear	France
Jacol et al. (41)	102, 009	all sites	54, 867	47, 142	possible fear	Germany
Coma et al. (42)	273, 379	all sites	72.8/10^5^	54.6/10^5^	Complex possible fear	Spain
Aparicio et al. (43)	3, 251	digestive	1, 866	1385	decreased endoscopy	France
Morris et al. (44)	64, 099	digestive	36, 274	27, 825	decreased endoscopy	England
Suarez et al. (45)	169	colorectal	111	58	complex possible fear	Multiple
Ranganathan et al. (48)	164, 030	all sites	112, 270	51, 760	lock down fear	India

No, number; COVID-2019, coronavirus disease 2019; NS, note specipied.

In a root cause analysis of the decreased emergency room visits during the pandemic, fear of the virus was the principal cause of the patients not going to the hospital even though they had life threatening medical conditions such as myocardial infarction or stroke. The hospital was seen as a reservoir for the virus with infection spreading through coughing and aerosol-generating procedures (49). In addition, there were concerns that the hospital did not take adequate measures to protect the patients from infection. Thus, it is reasonable to postulate that older patients who may have cancer would not seek medical attention due to the fear of death from SARS-CoV-2 infection. Therefore, any proposed solution to support older cancer patients should include measures to allay their fear.

## Impact of COVID-19 on cancer treatment

As a result of COVID-19 fear and/or decreased hospital admissions, the number of surgical procedures for cancer was decreased during the pandemic (38, 44). A survey of surgical oncologists across 69 European institutions revealed an average reduction of 29.3% for all types of oncologic surgery due to a reduction of outpatient clinic visits and a reduction of hospital beds or operating room availability (47). Of the physicians surveyed, 89.9% reported that fear of COVID-19 was the reason for the patients’ clinic appointment cancellations. Another study (50) reported that one of the main reasons for the reduced oncologic surgical procedures was the decrease on the number of endoscopic procedures performed in older cancer patients. Compared with the previous year, only 51% of older patients underwent diagnostic endoscopies. In addition, most of older individuals underwent palliative surgery rather than curative resection which may reflect their frailty status and/or it could reflect prioritizing younger patients for treatment (50). Other studies also corroborate the reduction in cancer surgery during the COVID-19 pandemic, and the pandemic was also associated with a significant delay in surgical treatment leading to a more advanced stage at diagnosis compared with pre-pandemic years (51–53). As a result, emergency surgery and non-curative resection were performed more frequently during the pandemic (54). For various reasons ranging from COVID-19 fear to delayed elective surgery, cancer surgery for all anatomic sites was significantly reduced (47, 50–56). It is postulated that the reduction in cancer surgery was related to decreased emergency room visits due to the widespread fear of the virus. However, the issue is more complex as patients may delay treatment, and this can lead to more severe symptoms and prolonged hospitalizations due to the severity of the disease (57, 58). As an ominous sign, the decrease and/or delay of surgery for cancer patients was also associated with a delay to adjuvant treatment such as chemotherapy which may further increase mortality rate (51).

The efficacy of systemic chemotherapy depends on adherence to the treatment. Any chemotherapy postponement may lead to disease recurrence. In a study of 3661 patients who received chemotherapy on an outpatient basis, the rates of chemotherapy postponement were 11.2% and 14.2% before and after the pandemic, respectively. In patients who had chemotherapy postponement, 17.4% stated that the reason was fear of COVID-19’s in light of their depressed immune system. Advanced age was the most common predictor of chemotherapy postponement (59). Importantly, among those who expressed fear and anxiety due to the virus, the introduction of telemedicine with patient education decreased the postponement rate to 4.6%, suggesting that this modality could be key to alleviating patients’ anxiety and improving treatment compliance. Other studies also reported a delay or modification of chemotherapy schedule, of up to 43.6% during the pandemic (60–66). In a study of 1472 cancer patients who received treatment at a tertiary cancer center, chemotherapy was delayed or discontinued in 51.6% and 12.6% of the patients, respectively (62). Of the patients who were showing a response to therapy before the pandemic, 10.3% had disease progression, and 73% of these patients died. Older age (60+), and disease progression were risk factors for death. More patients died from cancer progression than from COVID-19 infection. Another study also highlights the impact of oncology treatment interruption on survival. Among 112 head and neck cancer patients undergoing chemotherapy either alone or combined with radiotherapy, 71 discontinued treatment during the pandemic, and 31% of these showed disease progression or death (63). Even though many factors may have influenced treatment discontinuation, in one study of patients receiving adjuvant chemotherapy for early breast cancer, old age was a significant factor influencing the decision to discontinue treatment due to the fear of getting infected with SARS-CoV-2 while undergoing chemotherapy (66). Thus, all future policy should focus on avoiding treatment interruption, while also protecting patients from COVID-19 during treatment.

Radiotherapy treatment traditionally requires a course of five to seven weeks daily treatment to be curative depending on the cancer anatomic sites and histology. Delay or discontinuation of treatment may lead to a high risk of recurrence. Because of the necessity for long treatment time, it is inevitable that due to the prolonged lock down period, cancer patients may experience delayed and/or discontinued treatment. For example, in one study of 209 cancer patients, only 46.4% completed their radiotherapy course (67). In other studies, screening of patients for viral infection during treatment may also have led to unnecessary treatment interruption as patients with low grade fever are required to stay home as a safety measure (68). Up to 88.3% of the patients experienced radiotherapy delay or interruption due to various reasons (69). Many patients experienced a treatment break of at least 45 days (70). Coronavirus infection and fear of coronavirus infection also played a part in radiotherapy disruption (71, 72). **Table 2** summarizes cancer treatment delay or discontinuation during the pandemic.

**Table 2:** Decrease or delay in cancer treatment during COVID-19 pandemic.

Study	Treatment	Patient No.	Anatomic site	Pre-COVUD	Post COVID	Reasons	Country
Morris et al. (44)	Surgery	3381	colorectal	2003	1378	reduced endoscopy	England
Xu et al. (50)	Surgery	1538	colorectal	828	710	older patients at risk for infection reduced endoscopy	China
Li et al. (51)	Surgery	8357	breast	7075	1282	quarantine	China
Choi et al. (52)	Surgery	2901	colorectal	1985	916	unclear	Korea
Metzger et al. (53)	Surgery	624	colorectal	180	121	unclear	Germany
Okuyan et al. (54)	Surgery	301	colorectal	180	121	Delay of electivce Surgery, virus fear	Turkey
Guerrieri et al. (55)	Surgery	1248	genitourinary	720	528	Delay of elective surgery	Italy
Russel et al. (56)	Surgery	3889	all sites	2336	1553	Reduced surgical capacity	England
Karacin et al. (59)	Chemotherapy	3661	all sites	11.6% postponed	14.2% postponed	virus fear	Turkey
Sun et al. (60)	Chemotherapy	62	colon	unknown	50% postponed	hospital policy	China
Beypinar et al. (61)	Chemotherapy	159	lung col0orectal	3.7% postponed	39.8&	hospital policy Virus fear	Turkey
Valdiviezo et al. (62)	Chemotherapy	1828	all sites	unknown	51.6% postponed 10.3% discontinued	unclear	Peru
Chen et al. (63)	Chemotherapy	117	head and neck	unknown	60.6% discontinued	Lockdown	China
Gabor et al. (64)	Chemotherapy	146	gynecologic	unknown	24.6% modification	hospital policy patient cancellation	USA
Prabhash et al. (65)	Chemotherapy	514	Lung, CNS, urologic head and neck	unknown	16% modification	unclear	India
Gatfield et al. (66)	Chemotherapy	62	breast	unknown	16% discontinuation	Patient decision linked to old age	England
Xi et al. (67)	Radiotherapy	209	lung, breast, GI head and nech	unknown	53% discontinuation	lockdown	China
Lee et al. (68)	Radiotherapy	566	breast	6.6% interruption	8.2% interruption	COVID	Korea
Mitra et al. (69)	Radiotherapy	94	all sites	unknown	88% delay	COVID-19	India
Yu et al. (70)	Radiotherapy	140	all sites	unknown	100% discontinuation	lockdown	China
Rakici et al. (71)	Radiotherapy	195	all sites	unknown	4.6% discontinuation	COVID-19	Turkey
Koffler et al. (72)	Radiotherapy	NS	NS	8.1& disscontinuation	11.3% discontinuation	unclear	USA

COVID-19, coronavirus disease 19; no, number; NS, not specified.

It is clear that COVID-19 has affected early cancer diagnosis and has disrupted cancer treatment during the pandemic regardless of age, sex, or socio-economic status. However, the most affected were older cancer patients who, besides their fear of getting infected, also face other challenges in obtaining quality care such as social isolation, transportation difficulties, and age discrimination. While most cancer patients who contracted viral infection during the early phase of the pandemic only experienced a temporary interruption of their treatment, greater delays and modifications of treatment plans may have been considered necessary for older cancer patients (73). Thus, we propose a strategy combining modern technology such as telemedicine and spiritual support such as prayer, to improve the care of older patients during the COVID-19 pandemic, as it is very likely that the virus will remain with us in the foreseeable future.

## Potential impact of telemedicine to alleviate COVID-19 fear and for effective support of older cancer patients and their caregivers

To be effective, any measure that aims to improve older cancer patients’ quality of care should include their caregivers: spouses, children, grandchildren, or friends if the patient is single. Cancer patient caregivers have to coordinate all the physicians’ appointments into their work schedule, become patient advocates, provide emotional support to the patient, and manage the side effects of the treatment such as nausea and vomiting from chemotherapy. As a result, cancer caregivers develop a high level of anxiety and depression facing a task that they are unprepared for (74). In addition, minority patients often have a higher rate of depression than Caucasians, which is likely related to a lower socio-economic status (75). They also face transportation and communication barriers with health care professionals (76). Minority patients tend to instead rely on spirituality to overcome fears and find hope, to a greater extend than other ethnic groups (77, 78). Across the world, lower socio-economic status is also associated with a higher level of mental distress and depression similar to levels in minority patients. In the time of COVID-19, prayer and faith are the coping mechanisms for those patients. It seems logical to group them as economically disadvantaged (ED) patients to reflect the challenges that they face into obtaining quality of care.

The immediate impact of telemedicine is a reduced risk of SARS-CoV-2 infection while providing care to older cancer patients and their caregivers in the comfort of their home environment. Its real-time approach allows the patients and their caregivers active participation with the medical team which results in a high level of satisfaction (79). The Veterans Health Administration system, among the nation’s largest health care system, has successfully implemented telemedicine for cancer patients (80). Both medical oncologists and radiation oncologists can conduct preliminary telehealth visit with the patients and the caregivers. Surgical oncology consultation can also be conducted virtually for low-risk procedures or in cases that require only imaging for surgical planning. If the patient is suspected to have cancer, an electronic consultation is requested by the primary care physician. The case is then reviewed by an expert or discussed through a virtual tumor board. Biopsy and further work-up can be arranged afterward. This approach is innovative because it reduces the need for transportation for patients who live in rural areas far away from medical centers. The state of Alaska also delivers a telehealth network to provide education to Native and Alaskans cancer survivors that is tailored to their culture (81). Thus, telemedicine may improve the quality of life (QOL) of minority older cancer patients and their caregivers by focusing on special aspect of their culture such as spirituality as a support mechanism.

The first message by the medical team through virtual meeting should highlight the clinic or hospital safety measures to prevent coronavirus infection such as temperature check, screening for COVID-19 exposure, personal protection equipment (PPE), full vaccination of the medical staff, a COVID-19 testing policy, and strict adherence to a clean environment. If a patient tests positive for the virus, what are the measures taken to keep the other patients safe? Those are the two main concerns expressed during a survey by patients who were afraid to seek emergency care due to the fear of the virus (49).

Once the patient and the caregiver are reassured, the medical team should introduce themselves. Ideally, the medical team should include oncologists (medical, surgical, and radiation oncologist), a geriatrician, a patient navigator, a social worker, a home health nurse, a faith-based leader, and if needed a translator for non-English speaking patients. Many telemedicine platforms are now available to provide virtual consultation with multiple health care providers at the same time for patients and their family members who may live in different states. This will give the patients and their caregivers the confidence that they will be in charge with a professional team to assess all their needs from daily transportation to spirituality. This is particularly important for ED cancer patients who often feel that their spiritual needs are not supported by their health care providers (82).

The medical team should also emphasize that the side effects that the patient may experience during treatment such as nausea, vomiting, or pain can be managed through telemedicine to avoid unnecessary visits to the emergency room which would further expose them to the virus. Caregivers’ education is particularly important for older cancer patients due to their frailty and possible mental issues. Stress and anxiety should be assessed through questionnaires, and mental health referral should be initiated if needed. Patient spiritual needs can also be assessed through questionnaires. Once the patients and their caregivers are satisfied with the initial consultation, further in-person or virtual follow-up visits can be scheduled. If a high level of religiosity is expressed by the patients and/or their caregivers, further follow-up visits can also be arranged to take place virtually with the religious representative.

## Special consideration for economically disadvantaged patients and their caregivers

Compared with other socio-economic groups, ED cancer caregivers carry a higher burden in taking care of loved ones while facing many challenges such as transportation issues in getting to physician appointment or treatment sessions, lack of access to computers for disease information and education, and in the case of Latinos or other immigrant cancer patients, a language barrier (76, 83, 84). However, it has been reported that ED patients cope well with adversity through spirituality as they are traditionally very religious (77, 78, 82, 85, 86). Spirituality is defined as an individual’s sense of peace, purpose, and connection to others, and beliefs about the meaning of life. In simple terms, it is a holistic belief that there is more in life than what is perceived on a sensory and physical level that connects people and the universe together. Common spiritual themes include life after death, seeking non-material happiness, and compassion for others. This aspect of their care is often overlooked by health care providers who may not be aware of cultural diversity. Thus, involvement of faith-based leaders and bilingual patient navigators in addition to health care workers, may help older ED cancer patients and their caregivers navigate through the treatment journey and improve their QOL. In contrast to the healthcare setting prevalent in the United States, in the Eastern world, spirituality, prayer, and meditation are more widely acceptable practices amongst all strata of society. The practice of prayer, meditation, and yoga, as well as other forms of spirituality have shown a definite role in reducing anxiety and improving QOL in cancer patients (87, 88). We therefore believe that inclusion of faith-based leaders in patient care is likely to be welcomed.

There is a good chance that telehealth may become widely adopted by ED older cancer patients, their caregivers, and globally. Surveys among Latinos and Africans Americans in inner cities or rural areas reveal a high level of enthusiasm for participation in telemedicine. Immediate feedback, reduced waiting time, increased access to specialists and access to multiple medical opinions are among the advantages that have been reported (89, 90). As an illustration of the popularity of telemedicine, among 10,657 adults of multiple ethnicities, Latinos and Africans Americans had a significantly higher rate of telemedicine participation than Caucasian Americans (91). Fear of COVID-19 may have accounted for the highest rate of telemedicine use among African Americans (92). A virtual environment may also be more conductive to overcoming mistrust among ED patients about discrimination by the healthcare system (92). A positive message is particularly important to encourage the COVID vaccination rate among ED cancer patients (93).

Preliminary data supports the efficacy of spiritual therapy in improving QOL in cancer patients and their caregivers, across the world (94–98). As an illustration, a randomized study of 65 breast cancer patients who underwent radiotherapy reported that those who received spiritual therapy had significantly increased well-being as assessed by the cancer QOL C-30 and breast cancer specific questionnaire BR-23 compared with the control group (96). Even though the study was small, it highlights the impact of psychotherapy utilizing religious technique to empower the patient to attain a non-material understanding of self, universe, incidences and phenomena. Prayer reduces anxiety and help patients cope with their disease. Other studies also corroborate the importance of spiritual therapy to improve cancer patient QOL across different cultures (94, 96–98). Both cancer patients and caregivers benefit from spiritual therapy. In previous studies, cancer patients or caregivers who possess high spirituality level also enjoy better QOL, compared with those who do not, due to enhanced ability to cope with the severe distress induced by their disease (99–101). In these studies, those with a high level of spirituality are defined as those who feel the presence of God in their life and believe that God may protect and heal their suffering. This feeling was reinforced by reading of the scripture which included stories of miraculous survival in impossible circumstances (100). Thus, as ED cancer patients and their caregivers have a generally high level of spirituality, prayers conducted virtually by a faith-based leader should improve their QOL before, during, and after cancer treatment.

The feasibility of virtual based chaplaincy has been investigated in one study. In a survey of 711 cancer patients who were screened for distress level, 212 expressed a high level of spirituality. Out of those, 124 spoke on the phone with the chaplain. An in-depth survey about spirituality and chaplain intervention was conducted, and on this basis 41 patients were scheduled for further intervention either by phone (n=30), or in person (n=11) (102). Even though this study was small, it highlights efficacy of conducting spiritual therapy in a virtual mode. During the pandemic, virtual prayers were conducted successfully as a group through a Zoom or Telegram platform. Participants who experienced loneliness due to isolation and social distancing, expressed a sense of harmony and connection to others and God which helped them to cope with the pandemic stress (103). By incorporating a faith-based leader in the telemedicine team, it is feasible that cancer patients and their caregivers who express a high spiritual level may benefit from virtual spiritual therapy.

## Special considerations for vaccination of older cancer patients with hematologic malignancies

COVID-19 vaccination should be recommended for older cancer patients, and in particular minorities, due to their high risk of death if infected. However, vaccine hesitancy is highest among African Americans and Hispanics. Vaccination hesitancy was 41.6% and 30.2% for African Americans and Hispanics, respectively compared with 26.3% for adult Caucasians Americans (104). The reason for vaccination hesitancy is complex but includes mistrust of the US government among other reasons (93). Thus, it is critical to promote a public health campaign about the safety and efficacy of COVID-19 vaccine to increase vaccination rate in minority cancer patients. Among those who receive vaccination, the protection rate conferred by the vaccine is not known with certainty. Even when fully vaccinated with a second boost dose, seroconversion rate in cancer patients was 88% and 70% for patients with solid tumors and hematologic malignancies, respectively (105). However, other studies have reported a lower seroconversion rate for patients with hematologic malignancies following vaccination which may improve with a third or a fourth vaccine dose (106, 107). As those patients are at high risk of death if infected, they should avoid public exposure. The role of telemedicine is particularly important to monitor older patients with hematologic malignancies.They should take proper COVID-19 protection measures, such as social distancing and wearing an N-95 respiratory mask during clinic visits. They should also be reassured that many measures have been in place to protect them from virus exposure during their time in the clinic. Trust of the medical system is fundamental to patient adherence to treatment.

## Special consideration to promote a social-media_based public campaign to reduce COVID-19 vaccine hesitancy and increase early cancer diagnosis and treatment among older cancer patients and the economically depressed population

In 1999, Ambassador Reverend Andrew Young declared in public his diagnosis of early prostate cancer to encourage young African Americans to get regular prostate cancer screening. He was treated with surgery and remains cancer-free to this day. This same time of action is needed during the COVID-19 pandemic. Public figures should follow Ambassador Reverend Young’s example and promote the need for early cancer diagnosis, as cancer when caught early is curable due to advance in treatment technology. As an illustration, early stage non-small cell lung cancer has an excellent rate of local control and minimal complications among older and frail patients with stereotactic body radiotherapy who are not candidates for surgery (108). The public message should continue to allay the fear of COVID-19, be positive about the effectiveness of the vaccine, and target ED patients and older people to seek early diagnosis and cancer treatment. Most ED patients have poor knowledge of cancer symptoms and/or face barriers to access cancer screening (109, 110). Lack of education about precision medicine, unmet psychosocial needs, and financial burdens are frequently cited by ED cancer patients as barriers to accessing quality treatment (110). Thus, social media interventions if conducted properly, may be effective in reaching that specific demographic for education and reassurance that chronological age is not an impediment for cancer treatment. A meta-analysis of social media intervention reported that use of a social media platform may be an effective way to improve basic cancer knowledge, increase cancer screening rates, and alleviate psychological distress through social support (111). As an illustration, pictures of sun-damaged skin and testimony from cancer survivors are powerful ways to educate a large public audience about the risk of skin cancer and the benefit of sunscreen (112). However, it is important to have a social influencer from the same ethnic, cultural, and age group who speaks out in simple terms in order to modify the behavior of those targeted (113). Participation of individuals from diverse ethnic groups and cultures in social media such as YouTube or Facebook may be more effective because of the sense of connection between the social influencer and their followers. There are many social media sites available, but according to the Pew Institute survey, YouTube and Facebook are the ones most predominantly used by individuals aged 65 or older (https://www.pewresearch.org). The current trend is very encouraging: many celebrities have opened up about their cancer diagnosis on World Cancer Day 2022, such as Karim Abdul Jabar who had chronic leukemia in 2008 and recently at the age of 73 was diagnosed with prostate cancer. Actress Jane Fonda also communicated her diagnosis of non-Hodgkin’s lymphoma through Instagram at the age of 84 and has begun her chemotherapy. Her message was very positive and reflected confidence about a good outcome. We hope that more older women will be encouraged by her example to seek medical care early if cancer is suspected. The role of faith-based social influencers was illustrated in a study which encouraged ED patients to seek cancer screening. Out of 778 ED patients who were not aware of cancer risks and the needs for early cancer screening, over a third had discussed cancer screening with their primary care providers following enrollment (114). Collaboration between health communication professionals and faith-based leaders was critical in educating those patients to seek cancer screening, with potentially life-saving consequences. As an international research network dedicated to older cancer patients, minorities, and women who are frequently excluded from clinical trials, we also hope that social media will be used in the future to attract older cancer patients to participate in clinical trials. Their enrollment in prospective studies will be crucial to develop guidelines to manage this vulnerable population.

## Special consideration for hypofractionated radiotherapy for older cancer patients after the pandemic

During the pandemic, many professional groups have advocated the use of hypofractionated radiotherapy to shorten the treatment course of cancer patients and to reduce the risk of exposure to the SARS-CoV-2 virus (115, 116). Given the reduced mobility of older cancer patients, preexisting comorbidities, and/or frailty, this delivery of higher biological doses of radiation within a shorter treatment time may be particularly advantageous. The reduced need for daily transportation without sacrificing treatment effectiveness is an extra incentive for ED patients. As an illustration, a once a week treatment for six weeks has been reported to be well tolerated among 486 older breast cancer patients following surgery with excellent local control and survival (117). Thus, hypofractionated radiotherapy alone or associated with systemic therapy should be investigated in future prospective studies for older cancer patients (118).

## Perspective on the impact of COVID-19 on cancer mortality and future pandemics

It is clear that COVID-19 may increase the mortality rate of cancer patients regardless of age, due to delayed diagnosis and/or treatment. Many algorithms have been proposed to estimate the mortality risk following the pandemic which is currently far from over (119, 120). On the other hand, the pandemic also highlights the plight of older cancer patients who are frequently excluded from clinical trials (121). It is a paradox that although cancer prevalence increases with age, the number of older patients recruited in cancer clinical trials is low. As an illustration, in a review of 356 cancer trials, 67.7% either impose a strict upper age limit or a criterion on performance status which would reliably exclude older adults (122). Thus, public awareness and social media exposure may have a positive impact on clinical investigators, influencing them to review and reconsider those strict criteria for older cancer patients recruitment into clinical trials. Support and education through social media about the challenges facing older cancer patients may lead to innovative solutions to help them enroll in clinical trials, as older cancer patients may be socially isolated and less computer savvy than the younger generations (123, 124). In addition thanks in large part to the pandemic, the use of telemedicine has been accepted by the public, government, and the physician community as a convenient and potentially cost-saving measure to deliver quality care at a distance (125). Thus, on the positive side, COVID-19 has provided lessons on how to prepare and protect the most vulnerable segment of our society, older cancer patients, from future pandemics.

## Conclusions

Older cancer patients have suffered disproportionally during the pandemic. Their fear of death due to viral infection has led to a delay in early cancer diagnosis and treatment. We propose telemedicine combined with spiritual therapy and a strong message through social media, as a way to allay the fear of these patients. In addition, we propose the development of treatment strategies that take into consideration ethnicity and culture for personalized treatment.

## Data availability statement

The original contributions presented in the study are included in the article/supplementary material. Further inquiries can be directed to the corresponding author.

## Author contributions

All authors collected the data, discuss the data analysis, and review the final draft. All authors contributed to the article and approved the submitted version.
